# Contemporary outcome of the surgical management of prosthetic graft infection after a thoracic aortic replacement: is there a room to consider vacuum-assisted wound closure as an alternative?

**DOI:** 10.1007/s11748-014-0451-5

**Published:** 2014-07-20

**Authors:** Tomoyuki Suzuki, Shunsuke Kawamoto, Naotaka Motoyoshi, Masatoshi Akiyama, Kiichiro Kumagai, Osamu Adachi, Yukihiro Hayatsu, Koki Ito, Satoshi Matsuo, Yoshikatsu Saiki

**Affiliations:** Division of Cardiovascular Surgery, Tohoku University Graduate School of Medicine, 1-1 Seiryocho, Aoba-ku, Sendai, 980-8574 Japan

**Keywords:** Thoracic aorta, Graft infection, Vacuum-assisted wound closure

## Abstract

**Objective:**

Once a replaced prosthetic graft is infected, it is usually necessary to re-replace the thoracic aorta to achieve complete resolution of the infection. It is, however, an exceedingly invasive approach to perform such a repeat surgery on patients in a poor condition. We have managed both re-replacement of an infected prosthetic graft and conservative therapy with vacuum-assisted wound closure (VAC) without re-replacement. These two treatment modalities were retrospectively assessed.

**Methods:**

Retrospective clinical chart review was undertaken on 21 patients with prosthetic graft infection after thoracic aortic replacement between December 1999 and December 2012. Surgical outcomes were evaluated between the two groups: re-replacement group (group R, *n* = 14) and no-replacement group (group NR, *n* = 7).

**Results:**

In-hospital survival rates were 64.3 % in group R and 85.7 % in group NR. Mortality in group R included five patients, sepsis in two patients, and intraoperative aortic rupture, heart failure, and cerebral infarction in one. Mortality in group NR included one patient (sepsis). In terms of long-term outcome, one patient in group R and one patient in group NR died of rupture of a residual aortic aneurysm, and one patient in group NR died of renal disease during follow-up (52.8 ± 41.5 months for R and 43.2 ± 28.5 months for NR; mean ± standard deviation).

**Conclusions:**

Re-replacement of an infected prosthetic graft after a thoracic aortic operation still carries a significant risk for mortality. VAC therapy may provide an acceptable option for such a subgroup of patients with this serious condition.

## Introduction

Infection of a prosthetic graft after thoracic aortic surgical procedure has been a significant life-threatening risk for decades [1]. For such a condition, excision of the infected graft with drainage is thought to be a radical and reliable way to achieve complete resolution of the infection. Re-replacement of the infected graft with a homograft has been the first-line therapeutic protocol at our facility. Nevertheless, re-replacement is a great burden for severely infected patients, and occasionally, an unrealistic option even if the procedure per se is executable. There have been some reports from an early era that indicate the possibility to resolve the infection of the graft using drainage and minute irrigation without replacement [2–5]. This conservative therapy can be strengthened by introducing a vacuum-assisted wound closure (VAC) system and appears to be more effective with the latter system [6]. This non-invasive system removes microorganisms, inflammatory mediators, and slime out of the tissues and promotes granulation by maintaining continuous negative pressure. We have previously reported the effectiveness of the VAC system for resolution of infection in prosthetic grafts without detrimental dehiscence of anastomosis sites [6]. On the other hand, probability of complete resolution is still unknown for such conservative management of graft infection, and the method may not always be applicable to specific conditions such as infectious pseudoaneurysm formation. The contemporary outcomes of the surgical management of prosthetic graft infection need to be studied further. This retrospective clinical review was undertaken to evaluate two types of modalities for this lethal condition in our current clinical practice.

## Subjects

Between December 1999 and December 2012, 21 patients who were diagnosed with prosthetic graft infection after thoracic aortic replacement underwent two modalities of treatment. Cases of aorto-esophageal fistulae were excluded from the analysis because of their distinctive clinical features and unique staged treatment procedures. The diagnosis of prosthetic graft infection was made according to the overall findings such as a high fever, upregulation of inflammatory biochemical indicators, aberrant CT findings, and positive blood culture. Cases of shallow subcutaneous wound infection or osteomyelitis of the sternum without evidence of graft infection were also excluded from the analysis. The treatment strategies included re-replacement therapy of an infected prosthetic graft (group R, *n* = 14) and VAC therapy (no-replacement group, i.e., group NR, *n* = 7).

## Methods

The perioperative and follow-up data on the 21 patients were based on the retrospective clinical chart review and the reference to neighboring hospitals under patient consent at the operation. The perioperative characteristics and clinical outcomes were reviewed and compared.

### Selection of a treatment modality

The decision on which method (re-replacement or VAC) should be used in each case depended upon multiple factors including a patient’s general condition. When a pseudoaneurysm was present, the VAC system installation was not adequate. An apparent intra-graft development of vegetations also precluded VAC therapy, and re-replacement of the graft was performed instead. Lateral thoracotomy infection was not a good indication of VAC therapy either. In both groups (R and NR), broad-spectrum antibiotics were initially administered and adjusted according to the culture results.

### The re-replacement procedure (group R)

In group R, the chest was opened for drainage and irrigated with saline followed by immediate re-replacement of the infected graft in six patients or the mediastinum was irrigated (or packed with povidone-iodine bond gauze in early years) once a day for 2–31 days [12.8 ± 11.6 (mean ± standard deviation), same as below] to reduce the amount of bacteria prior to a re-replacement surgical procedure in eight patients. As described above, an aortic homograft was the choice of the first-line substitute when available; otherwise, a rifampicin-bond prosthetic graft was used for graft replacement.

### The VAC procedure (group NR)

**Fig. 1:**
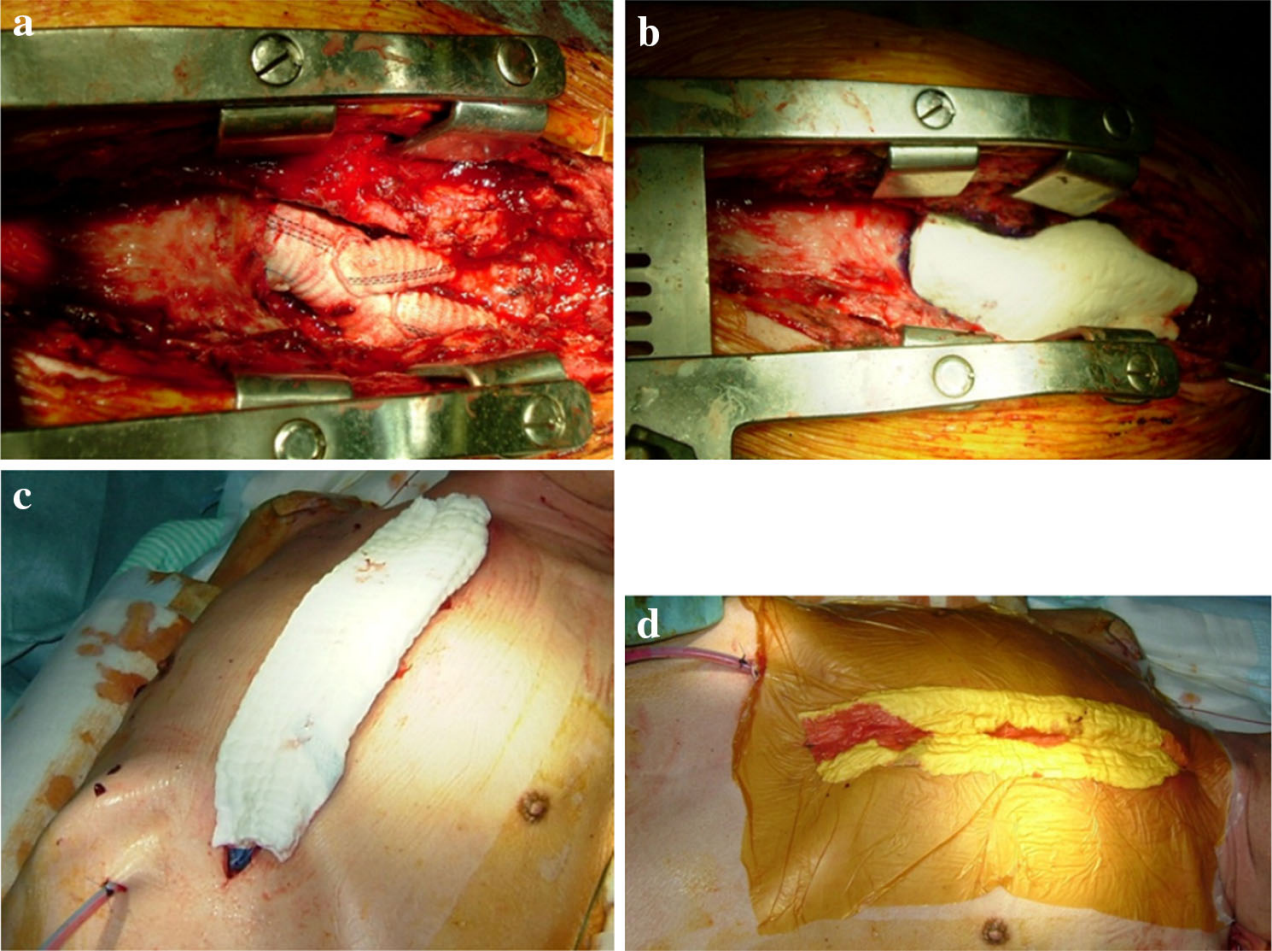
**a** The chest is opened and the infected graft is irrigated, **b** the anastomosis and the surface of heart are covered with polyurethane sponge, **c** uncollapsible chest tube is placed over the sponge, and the wound is covered with towels, **d** the whole wound is covered with surgical drape, and the negative pressure is generated through the tube

In group NR, the VAC system was installed as described elsewhere [6]. In brief, after the exploration and drainage of the chest, polyurethane sponges were sheeted to cover the entire surface of the mediastinum. This sponge was necessary for protection of the anastomotic site or the surface of the heart from direct negative pressure and for generation of uniform negative pressure in the whole treatment area. A chest tube was placed over the sponge, and the wound was covered with towels and surgical drapes. Then, negative pressure of −99 mmHg was generated by means of a vacuum pump (HAMA Servo drain, Hama Medical Industrial Co., Ltd., Tokyo; Fig. 1). We had been applying this device before the KCI^®^ apparatus became available in Japan, and this pressure is maximal for this device. Exchange of the whole system, including the polyurethane sponges and routine irrigation, was performed once a day at least for the subsequent several days. Closure of the chest was undertaken after confirming negative bacterial culture in the wound.

### Statistics

All calculations were performed using the SPSS software (SPSS, Inc., Chicago, IL, USA). Continuous variables were expressed as mean ± standard deviation and compared using the *t* test. Categorical variables, expressed as percentages, were analyzed using either the Chi squared or Fisher’s exact tests. Survivals in the two groups were compared using the Kaplan–Meier method. A *p* value less than 0.05 was assumed to indicate statistical significance.

## Results

The age was 56.4 ± 21.1 years (range 7–78 years) in group R and 70.9 ± 10.0 years (range 52–78 years) in group NR. Pre-operative patients’ demographics in each group are shown in Table 1. Characteristics of the patients in the two groups were essentially the same: there were no statistically significant differences in the pre-operative factor.

**Table 1:** Pre-operative patients’ demographics and comorbidities in each group

	Group R (n = 14)	Group NR (n = 7)	*p* value
Age	56 (7–78)	71 (52–78)	0.106
Male	11 (78.6 %)	4 (57.1 %)	0.299
Prior operation			
Emergent	8 (57.1 %)	3 (42.9 %)	0.438
Re-sternotomy	5 (35.7 %)	1 (14.3 %)	0.314
Diabetes mellitus	1 (7.1 %)	1 (14.3 %)	0.567
Dialysis	1 (7.1 %)	0	0.667
Creatinine (mg/dL)	1.14 (± 1.13)	0.79 (± 0.29)	0.428
COPD	3 (21.4 %)	1 (14.3 %)	0.593
Medication			
Immunosuppressant	0	1 (14.3 %)	0.333
Steroids	0	1 (14.3 %)	0.333
Heart failure	3 (21.4 %)	0	0.274
CRP (mg/dL)	11.9 (± 9.0)	10.8 (± 14.9)	0.834

*COPD* chronic obstructive pulmonary disease, *CRP* C-reactive protein

Perioperative factors and surgical outcomes are summarized in Table 2 for group R and Table 3 for group NR. Five of seven patients in group NR were already reported in our previous article [6]; the updated outcomes are included in Table 3.

**Table 2:** Perioperative status, procedures, and outcomes in group R

No	Age	Sex	Prior surgery	Interval	Culture organism	Perioperative management	Second surgery	Organism at closure	Hospital survival	Follow-up
1	51	M	Root+arch repl.	4 months	MRSA	Exploration 14 days of drainage and irrigation	Root+arch repl. (homograft + latissimus dorsi muscle flap)	MRSA	Dead (CVD, POD 19)	–
2	70	M	Descending repl.	7 months	MRSA	Exploration 3 days of povidone iodine gauze packing	Descending repl. (homograft)	MRSA	Survived	114 months
3	7	M	MVR, RV-PA repair	10 months	MRSA	Exploration 2 days of drainage and irrigation	PA reconst. (homograft + omentopexy)	Negative	Survived	47 months
4	65	M	Arch repl. aortic valve suspension	27 months	Anaerobic bacteria	Exploration 2 days of povidone iodine gauze packing	Arch repl. (homograft + omentopexy)	Negative	Survived	59 months (dead: TAAA rupture)
5	73	F	Ascending repl.	9 months	α Hemolytic Streptococci	Exploration 31 days of drainage, irrigation, and VAC	Ascending repl. (homograft + omentopexy)	*Pseudomonas aeruginosa*, *Stenotrophomonas maltophilia*	Dead (LOS, POD8)	–
6	60	M	Root+proxmal arch repl.	14 months	*Staphylococcus epidermidis*, GNF-GNR	None	Root repl. (stentless valve + omentopexy)	Negative	Survived	103 months
7	78	M	Descending repl.	24 months	*Pseudomonas aeruginosa*	Exploration 4 days of drainage and irrigation	Arch repl. (homograft)	*Enterococcus faecium* *Pseudomonas aeruginosa*	Dead (intraoperative rupture)	–
8	19	M	AVR with annular enlargement	4 months	MRSE	None	Ross operation (omentopexy)	Negative	Survived	89 months
9	68	M	Descending repl.	43 days	MRSA	Exploration after stent grafting 24 days of drainage and irrigation	① Stent-graft ② Descending repl. (homograft + omentopexy + rectus abdominis muscle flap)	MRSA	Dead (sepsis, POD7)	–
10	67	F	TAAA repl.	37 days	MRSA	Exploration 22 days of drainage and irrigation	TAAA repl. (homograft + omentopexy)	MRSA *Klebsiella pneumoniae*	Dead (sepsis, POD20)	–
11	56	M	AVR ascending repl.	41 months	Negative	None	Ascending repl. (homograft + omentopexy)	Negative	Survived	38 months
12	75	F	Ascending repl.	15 months	*Candida albicans*	None bofore operation 9 days of VAC after the operation	① Root and ascending repl. + VAC (stentless valve + refampicin-bond graft) ② Closure + omentopexy	Negative	Survived	8 months
13	62	M	Arch repl.	159 months	*Streptcoccus mitis* group	None	Root repl. (refampicin-bond graft)	*Streptcoccus mitis* group	Survived	9 months
14	39	M	Ascending ~ arch ~ descending repl.	47 months	*Streptococcus sanguis*	None	① Splenectomy ② Descending repl. (refampicin bond graft + omentopexy)	Negative	Survived	8 months

*repl* replacement, *MVR* mitral valve replacement, *RV* right ventricle, *PA* pulmonary artery, *AVR* aortic valve replacement, *TAAA* thoracoabdominal aortic aneurysm, *MRSA* methicillin-resistant *Staphylococcus aureus*, *GNF-GNR* glucose-nonfermentative Gram-negative bacilli, *MRSE* methicillin-resistant *Staphylococcus epidermidis*, *VAC* vacuum-assisted wound closure, *CVD* cerebro-vascular disease, *POD* post-operative day, *LOS* low cardiac output syndrome

**Table 3:** Perioperative status, procedures and outcomes in group NR

No	Age	Sex	Prior surgery	Period before VAC	Culture organism	VAC therapy	Second surgery	Mediastinal culture	Hospital survival	Follow-up
1	77	M	Arch repl.	5 days	*Enterobacter cloacae*	11 days	Closure + omentopexy	Negative	Survived	67 months (dead: renal disorder)
2	78	M	Arch repl. CABG	72 months	*Candida parapsilosis*	9 days	Closure + omentopexy	Negative	Survived	45 months
3	78	M	Arch repl, BCAR	3 months	MRSA	55 days	Debridment + closure + pectoral muscle flap + omentopexy	Negative	Survived	16 months (dead: TAAA rupture)
4	52	F	Root + arch repl.	48 months	MSSA	12 days	Closure + omentopexy	Negative	Survived	84 months
5	76	F	Hemiarch repl. root repl. re-root repl.	1 month	MRSA *Klebsiella*	26 days	–	Negative	Dead (sepsis, VAC day 27)	–
6	62	F	AVR,MVP, ascending ~ arch repl.	14 months	MSSA	15 days	Closure + omentopexy	Negative	Survived	36 months
7	73	M	Ascending repl.	① 8 months ② 4 months	MRSA	① 27 days ② 29 days	① Closure + omentopexy ② Closure + abdominal rectus muscle flap	① Negative ② Negative	Survived	11 months

*VAC* vacuum-assisted wound closure, *repl.* replacement, *CABG* coronary artery bypass grafting, *BCAR* brachio-cephalic artery replacement, *AVR* aortic valve replacement, *MVP* mitral valve plasty, *MRSA* methicillin-resistant *Staphylococcus aureus*, *MSSA* methicillin-sensitive *Staphylococcus aureus*

As a substitute for an infected prosthetic graft, an aortic homograft was used for re-replacement in nine cases (64.3 %) and another prosthetic graft in the remaining five cases (35.7 %) including when it was used as a supplemental material during the Ross procedure. Technical modifications in group R included a pre-operative use of the VAC system for case No. 5 in an attempt to reduce the amount of bacteria prior to a re-replacement operation and a postoperative installation of the VAC system immediately after a re-replacement procedure in case No. 12 until the chest was eventually closed.

### Deaths and recurrence in group R

There were five in-hospital deaths in group R, and one of the hospital survivors died of a ruptured dissecting descending thoracic aortic aneurysm during follow-up. The mean duration of follow-up was 52.8 ± 41.5 months. In particular, patient No. 1 died of a stroke on postoperative day (POD) 19 after re-root and arch replacement, and patient No. 5 developed low cardiac output syndrome with uncontrolled systemic infection and died on POD 8 after the re-replacement of an ascending aortic graft. Patient No. 7 died of an intraoperative rupture of the distal aortic arch during the procedure with a homograft. Patient No. 9 died of sepsis on POD 7 after re-descending aortic replacement, and patient No. 10 also died of sepsis on POD 20 after re-thoracoabdominal aortic replacement. At follow-up, the wound in case No. 8 became erosive and tested positive for methicillin-resistant *Staphylococcus aureus* (MRSA) 10 months after the re-replacement surgery. His mediastinum was then re-explored for VAC therapy. As a result, three patients died in the hospital because of systemic infection in spite of thorough replacement of the graft.

### Deaths and recurrence in group NR

In group NR, patient No. 5 died of sepsis on the 27th day after installation of the VAC system. With regard to the long-term outcome, patient No. 1 died of renal disease during the follow-up of 67 months, and patient No. 3 died of a residual-aneurysm rupture during the follow-up of 11 months. The mean follow-up period in group NR was 43.2 ± 28.5 months. Infection of the same site was recurrent only in case No. 7 of group NR. This patient had undergone a replacement of the ascending aorta for acute aortic dissection elsewhere. He developed the graft infection several months later and was treated with VAC therapy at another hospital. He developed recurrent mediastinitis and was thereafter transferred to our institution. His mediastinum was re-explored, and subsequently the VAC system was re-installed. The sternum had been almost debrided at the previous hospital; therefore, the skin was re-approximated after resolution of the mediastinitis. Unfortunately, he developed third-time mediastinitis associated with the graft infection 4 months later. We re-installed the VAC system and continued this therapy for 29 days, and his chest was closed using the rectus abdominis flap technique. After the final chest closure, there has been no sign of recurrent infection during follow-up.

The median interval between a previous procedure and re-replacement or VAC installation for graft infection was 12.0 months in group R and 8.0 months in group NR. The median duration of VAC therapy was 15.0 days.

The in-hospital survival rates of group R and group NR were 64.3 and 85.7 %, and 5-year survival rates according to the Kaplan–Meier analysis were 48.2 and 68.6 %, respectively.

## Discussion

It has long been assumed that a fundamental treatment strategy should include re-replacement of the infected prosthetic graft with an in situ substitute or an extra-anatomical bypass graft [7–10]. Conversely, graft-preserving conservative therapy has also been reported in the literature. Conservative treatment modalities for a prosthetic graft infection in the peripheral area were reported as early as 1963 [2], and a case treated with such a therapy for an abdominal aortic lesion was reported in 1991 [3]. As for thoracic aortic lesion, some successful treatments of the infected vascular grafts without a removal were reported in 2001 and 2007 [4, 5]. Recently, the effectiveness of VAC therapy against refractory wound infection has been proven, and its indication has also been extended to mediastinitis [11–14]. Nonetheless, there have not been many reports describing liberal use of VAC therapy for prosthetic graft infection after a thoracic aortic surgical procedure. Furthermore, a long-term outcome after VAC therapy for that condition has never been determined. In this study, we worked on both re-replacement of an infected prosthetic graft and conservative therapy with the VAC system without re-replacement. We found that re-replacement of the infected prosthetic graft after a thoracic aortic operation still carries a significant risk for mortality. VAC therapy yielded favorable outcomes in a subgroup of patients with this serious condition, although the two treatment modalities were assessed only retrospectively; therefore, a selection bias for each procedure cannot be ruled out.

An adjuvant therapy was employed at the time of the chest closure in all the cases in group NR. In other words, the coverage of the prosthetic grafts and obliteration of a mediastinal dead space using an omentum or muscle flaps may be crucial for long-term prevention of recurrent infections [15]. The successful management for thoracic aortic graft infections using the omental pedicle was reported by Miller et al. [16] in 1987. In their report, the usefulness of omentum included the ability of absorbing excessive fluid by way of rich vasculature and lymphatic channels and provision of adequate mass volume to fill up some dead space. Subsequently, applications of omental flap were extended for the mediastinitis and difficult thoracic wounds [17, 18]. Based on these documented reports, we elected to apply omentopexy as an adjuvant therapy to minimize the risk of recurrent infection, even though mediastinal wound cultures were confirmed to be negative before sternal closure in our series.

Although the VAC system was proven as safe and effective, this method cannot be used in all cases. When we encounter a pseudoaneurysm or intra-graft vegetation after re-exploration, adequate surgical replacement is unavoidable. For a lateral thoracotomy incision site, installation of the VAC system for the infected prosthetic graft is difficult because of the depth of the lesion, width of the pleural space, friability of lung tissue, and the difficulty in maintaining an adequate physical position throughout postural changes. Hence, each case has to be individually considered whether it can be treated conservatively with the VAC system, or what kind of drainage or surgical procedure and to what extent the graft re-replacement should be performed. Even nowadays, adequate selection of strategy according to a patient’s individual status is necessary when little evidence is available to guide surgeons in treating these patients [19].

## Limitations

There were different numbers of cases in the groups and a relatively small total sample size. In addition, the VAC system could be applied only to selected cases; consequently, the backgrounds of the patients in the two groups are different.

## Conclusion

The prognosis of a prosthetic graft infection after a thoracic aortic procedure is not yet satisfactory, but compared with surgical re-replacement, VAC therapy is an acceptable and less invasive method for resolving this challenging situation. Although selection of cases and appropriate conditions for installation of the VAC modality are necessary, it can be considered a reasonable option to achieve complete resolution of a life-threatening infection.
